# Attachment of DNA-Wrapped Single-Walled Carbon Nanotubes (SWNTs) for a Micron-Sized Biosensor

**DOI:** 10.1021/acsomega.2c06278

**Published:** 2022-12-09

**Authors:** Kota Hirayama, Masaki Kitamura, Nay San Lin, Minh Hieu Nguyen, Binh Duong Le, Anh Tuan Mai, Shigeki Mayama, Kazuo Umemura

**Affiliations:** † Biophysics Section, Department of Physics, Faculty of Science Division II, Tokyo University of Science, 1-3 Kagurazaka, Shinjuku, Tokyo 162-8601, Japan; ‡ VNU University of Science, 334 Nguyen Trai, Thanh Xuan, Hanoi 10000, Vietnam; § National Center for Technological Progress, 25 Le Thanh Tong, Hoan Kiem, Hanoi 100000, Vietnam; ∥ VNU University of Engineering and Technology, 144 Xuan Thuy, Cau Giay, Hanoi G2-206, Vietnam; ⊥ Tokyo Diatomology Lab, 2−3-2 Nukuikitamachi, Koganei, Tokyo 184-0015, Japan

## Abstract

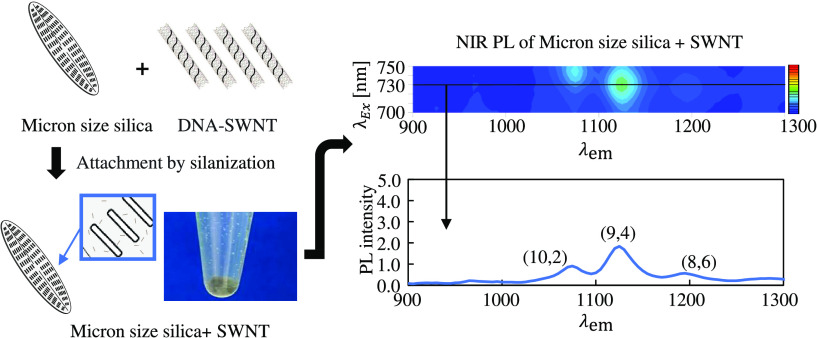

We fabricated a micron-sized biodevice based on the near-infrared photoluminescence (PL) response of single-walled carbon nanotubes (SWNTs). Various biosensors using the unique optical responses of SWNTs have been proposed by many research groups. Most of these employed either colloidal suspensions of dispersed SWNTs or SWNT films on flat surfaces, such as electrodes. In this study, we attached DNA-wrapped SWNTs (DNA-SWNTs) to frustule (micron-sized nanoporous biosilica) surfaces, which were purified from cultured isolated diatoms. After the injection of an oxidant and a reductant, the SWNTs on the frustules showed prominent PL responses. This suggests that the biodevice functions as a micron-sized redox sensor. Frustules can be easily suspended in aqueous solutions because of their porous structures and can easily be collected as pellets by low-speed centrifugation. Thus, the removal of unbound SWNTs and the recovery of the fabricated DNA-SWNT frustules for reuse were achieved by gentle centrifugation. Our proposal for micron-sized SWNT biodevices would be helpful for various biological applications.

## Introduction

The unique optical responses of single-walled carbon nanotubes (SWNTs) in the near-infrared (NIR) region make them suitable for use in various biosensing applications.^1−3^ For example, when an oxidizing agent is added to an SWNT suspension, the NIR absorbance and NIR photoluminescence (PL) of SWNTs generally decrease.^4−6^ If a reducing agent is added, the opposite reactions are observed, although there are exceptions to this. The optical responses are sensitive; therefore, the NIR spectra of SWNTs can fluctuate even when the pH of the suspension is changed.^7−9^ Recently, Card et al. induced the optical responses of an SWNT embedded in a hydrogel film by adding calcium chloride.^10^ The antioxidant abilities of biomolecules can be quantitatively analyzed by using SWNTs.

The chirality of SWNTs is an important parameter for obtaining multichannel information from the optical responses of SWNTs.^11−13^ Chirality, which is defined by the chirality vector (*n*, *m*), determines the structural and physicochemical properties of SWNTs. For example, metal and semiconductor SWNTs can be categorized according to (*n*, *m*) values.^14^ The sensitive optical responses are obtained from semiconductor SWNTs.^15−17^

One of the basic and key techniques to establish the applications of biosensing is the solubilization of isolated SWNTs.^18−20^ The specific optical responses appear when individual SWNTs are isolated. Many biological applications have been realized in aqueous solutions. However, bare SWNTs are not water soluble and form bundles. Bundled SWNTs do not exhibit sensitive optical responses.

Wrapping techniques of SWNTs with surfactants and organic molecules, including biomolecules, have been widely used to prepare SWNT suspensions.^18−23^ For example, DNA-wrapped SWNTs (DNA-SWNTs) achieve stable aqueous suspensions.^18−20^ Although double-stranded DNA (dsDNA) can be used for this purpose, single-stranded DNA (ssDNA) provides more stable conjugates with SWNTs. SsDNA is an amphipathic molecule because the four bases and the phosphate backbones of ssDNA molecules are hydrophobic and hydrophilic, respectively.^20^ In dsDNA, the bases are embedded within the double helix, whereas in ssDNA they are exposed.^24^ Various theoretical models have suggested that the exposed DNA bases have a high affinity for bare SWNTs.^25−27^ When SWNTs are bound to the bases of ssDNA molecules, the hydrophilic phosphate backbones are at the surface of the conjugates. In this approach, DNA and SWNTs are bound via physisorption. The resonance structures of SWNTs are preserved completely, and the above-mentioned sensitive optical responses of SWNTs remain unaffected. Therefore, DNA-wrapped SWNTs (DNA-SWNTs) have been widely used in biological applications, such as the detection of DNA hybridization and the measurement of glucose concentrations.^17,28,29^

Another powerful technique in the development of biodevices is the use of micron-sized objects, such as microbeads. Many studies have reported the attachment of enzymes or DNA onto microbead surfaces. By the attachment of water-soluble biomolecules to micron-sized surfaces, biodevices can be recovered. For example, Venezia et al. attached glucosidase enzymes to mesoporous silica surfaces to develop a glucose sensor.^30^ After demonstrating enzyme reactions using the micron-sized glucose sensor, the sensors were easily recovered by centrifugation. Without the attachment to the micron-sized silica, it would have been difficult to recover the enzymes after the reaction.

For carbon nanotubes (CNTs), there are many reports that describe attachment of CNTs to electrode surfaces.^31,32^ However, this approach uses electrochemistry as the main method for detecting biological reactions on the electrode surfaces and employs multiwalled carbon nanotubes (MWNTs), which do not exhibit PL.

For biosensing applications using the PL of SWNTs, only a few studies have reported the attachment of SWNT conjugates to solid surfaces. Shumeiko et al. deposited peptide-wrapped SWNTs on a paper surface to create protease sensors.^33^ They succeeded in detecting protease activities as a change in the PL spectra of SWNTs. Habimana et al. attached functionalized MWNTs to microbead surfaces to detect enzyme activity.^34^ In their case, the detection of enzyme reactions was facilitated by using appropriate dyes, not PL from CNTs. The recovery of biodevices by centrifugation was also demonstrated. However, the attachment of SWNT conjugates to micron-sized objects is a novel approach that has not been reported yet.

In this study, we attached DNA-SWNT hybrids to frustule surfaces using electrostatic forces. Frustules are nanoporous structures made of silica, which can be purified from living diatoms, one of the major components of phytoplankton. Unlike microbeads, which rapidly settle in aqueous solutions, frustules can float for a long time because of their small specific gravity (around 1.3). Our study is the first one to attach DNA-SWNT hybrids to micron-sized objects. We demonstrated the potential of this novel micron-sized device to detect the reaction of oxidants and reductants by photoluminescence.

## Methods

SWNT powders produced by the high-pressure carbon monoxide process (HiPco) were used (HS27-122, Raymor Industries Inc., Boisbriand, City, QC, Canada). SsDNA was 30-mers of thymine (10336022, Thermo Fisher, Massachusetts). Thymine is known to have a higher binding affinity with SWNTs than other bases.^35^ A potassium permanganate solution (42000375, Hayashi Pure Chemical Ind., Ltd., Osaka, Japan) was used as the oxidizing agent. Dithiothreitol (DTT, 040-29223, FUJIFILM Wako Pure Chemical Co., Osaka, Japan) was dissolved in 10 mM phosphate-buffered saline (PBS) to prepare a reducing agent. Catechin (E0694, Tokyo Chemical Industry Co., Ltd., Tokyo, Japan) was dissolved in 10 mM PBS and used as a reducing agent.

*Navicula* sp. diatom cells were collected from Chiba Prefecture, Japan. Isolated *Navicula* sp. cells were cultured in Guillard’s (F/2) culture medium (G9903; Sigma-Aldrich, Munich, Germany) with Daigo artificial seawater (395-01343; Nihon Pharmaceutical, Tokyo, Japan).^36,37^ Frustules from diatom cells in suspensions were treated with nitric acid. The cell suspensions were replaced with pure water by centrifugation (1630*g*, 5 min, five times). Diatom cells from 9 L cultures were suspended in approximately 100 mL of pure water. A quantity of concentrated nitric acid that was twice the volume of the cell suspension was added to remove any organic components. The mixture was then incubated for 40 min at 95 °C. To stop the reaction, potassium nitrate (a cupful of a medical spoon) was immediately added to the cell suspension and diluted with pure cold water on ice. Then, the nitric acid was removed by centrifugation (1630*g*, 5 min, seven times).

For the preparation of the DNA-SWNT suspension, a mixture of a ssDNA solution (10 mM PBS, pH 7.0) and SWNT powder (concentrations: ssDNA 1.0 mg/mL, SWNT 0.5 mg/mL) was sonicated for 90 min with an amplitude of 60%, a frequency of 20 kHz, and a power of 130 W at 0 °C. The sample was then centrifuged at 15,000 rpm (20,128*g*) for 180 min at 0 °C. Approximately 70% of the supernatant was stored as a DNA-SWNT suspension.

To attach DNA-SWNT hybrids to frustule surfaces, frustules were pretreated with 3-(2-aminoethylaminopropyl)trimethoxysilane (APTES, LS-2480, Shin-Etsu Chemical Co., Ltd., Tokyo, Japan). Approximately 500 μL of the mixture of frustules (Absorbance 0.5 at 750 nm) and APTES (final concentration 1%) was incubated for 1 h at room temperature under gentle rotation. The mixture was centrifuged at 5000*g* for 5 min at room temperature. Then, 80% of the supernatant was replaced with pure water to remove excess APTES (AP treatment). The supernatant was replaced by centrifugation, which was repeated five times. The DNA-SWNT suspension was then added to the functionalized frustule suspension (Absorbance of DNA-SWNT at 808 nm was adjusted to 0.2, and the turbidity of the prepared frustules at 750 nm was 0.5). The mixture was incubated for 1 h under gentle rotation at room temperature, followed by the removal of excess DNA-SWNT hybrids. The sample was centrifuged at 5000*g* for 5 min at room temperature, and 80% of the supernatant was replaced with 10 mM PBS to remove excess ssDNA-SWNTs, and this was repeated five times. The supernatants were stored for evaluation. Absorbance and turbidity were measured using a UV–vis spectrometer (V-630, JASCO, Tokyo, Japan). The amounts of SWNTs attached to the frustule surfaces and of SWNTs remaining in the supernatant were estimated by turbidity measurements at 808 nm.

For the PL measurements, an NIR-PL system (Shimadzu Co., Ltd., Kyoto, Japan) was employed. The light source was a 500 W xenon lamp, and the detector was an InGaAs sensor. The exposure time was set to 20,000 ms. PL map measurements were performed to evaluate the DNA-SWNT functionalized frustules. The excitation and emission wavelengths were 700–750 and 900–1300 nm, respectively. The exposure period was 20,000 ms/1 nm. The frustule suspension (250 μL) with attached DNA-SWNT hybrids was mixed with 250 μL of glycerol in a cuvette (suspension including 10 mM PBS and 50% glycerol). The absorbance of the frustules was measured before attaching DNA-SWNT hybrids. Frustules with attached DNA-SWNTs were centrifuged, and supernatants were replaced five times during silanization and SWNT adsorption. Therefore, the turbidity of frustules with attached DNA-SWNTs at 750 nm may be smaller than that of the initially prepared frustules. Glycerol was added to avoid frustule settlement during PL map measurements. For the purpose of comparison, DNA-SWNT suspensions without frustules were also evaluated using PL map measurements. Before and after the PL map measurements, a cross line of only 730 nm was measured to confirm that there were no settlements of frustules. The measurements were repeated five times using independent samples.

The PL responses of frustules with DNA-SWNTs in the presence of an oxidant and a reductant were examined by PL spectroscopy at an excitation wavelength of 730 nm. The emission wavelengths ranged from 900 to 1300 nm. The exposure period was 20,000 ms. The frustule suspension (500 μL) with attached DNA-SWNT hybrids was injected into a cuvette (suspension including 10 mM PBS). Glycerol was not added for the measurements this time to enable a smooth mixing of the added chemicals. For comparative purposes, DNA-SWNT suspensions without frustules were also evaluated. The cuvette was shaken well immediately before the measurements. The samples were first analyzed without any chemicals. After the first measurement, the same sample was remeasured as soon as possible without shaking to evaluate the settlement of frustules during the first measurement. Then, 5 μL of potassium permanganate (KMnO_4_) aqueous solution (final concentration: 5 μM) was added to the cuvette. After incubating for 10 min at room temperature, the sample was shaken again and measured as soon as possible. To evaluate the settlement of frustules, the same sample was measured twice. Finally, 5 μL of dithiothreitol (DTT) aqueous solution (final concentration: 50 μM) was added to the sample. After incubating for 10 min at room temperature, the sample was shaken again and measured as soon as possible. To evaluate the settlement of frustules, the same sample was measured twice. The measurements were repeated five times using independent samples.

In addition, the supernatant of the fifth centrifugation of the DNA-SWNT frustules was evaluated by spectroscopy at an excitation wavelength of 730 nm. The emission wavelengths ranged from 900 to 1300 nm. The exposure period was 20,000 ms.

In the reusability experiment, 500 μL of DNA-SWNT frustule suspension was recycled eight times to measure the redox reaction. The absorbance of the DNA-SWNT frustules at 808 nm was adjusted to 0.2, and the turbidity of the DNA-SWNT frustules at 750 nm was 0.5. KMnO_4_ (final concentration: 5 μM) was used as an oxidant, and DTT (final concentration: 50 μM) was used as a reductant. The DNA-SWNT frustule suspensions were measured by PL. The same suspensions were then measured after injecting KMnO_4_ (final concentration: 5 μM) and DTT (final concentration: 5 μM) and incubating for 10 min at room temperature. After completing the first measurement cycle, the DNA-SWNT frustule suspensions were recovered by centrifugation, and the supernatants were replaced. The DNA-SWNT frustule suspensions were made up to 1000 μL in PBS buffer. Then centrifuged five times at 5000*g* for 5 min, and 80% of the supernatant was replaced with PBS buffer. The recovered DNA-SWNT frustules were then reused. This cycle was repeated eight times.

Scanning electron microscopy (SEM; S4800, Hitachi, Ltd., Tokyo, Japan) was performed on frustules with DNA-SWNT hybrids. Frustules with DNA-SWNT hybrids were dropped on the carbon tape in the cleanroom. The sample was then dried at 20 °C for 2 h in the cleanroom. The sample covered a gold nanothin film on the surface of frustules.

Raman spectroscopy (RAMANtouch 11i VIS–NIR, Nanophoton Corporation, Osaka, Japan) was performed for SWNT powder, DNA-SWNT suspension, and frustules with DNA-SWNT hybrids. A small amount of SWNT powder was fixed on a glass surface using double-sided tape. Twenty microliters of the DNA-SWNT suspension, frustules with DNA-SWNT hybrids, and frustules were deposited on a glass surface and dried for 1 h using a vacuum dryer. The point mode was employed with an exposure period of 20 s. The excitation wavelength was 532 nm. The objective lens used was 20× magnification.

An atomic force microscope (AFM; MFP-3D microscope, Asylum Research, Santa Barbara, CA) was employed for the structural analysis of the DNA-SWNTs on the frustules. Experiments were conducted in AC-AFM mode and phase mode in air with the application of a silicon cantilever PPP-NCSTR-W (NANOSENSORS, Nanoworld AG, Neuchatel, Switzerland). Mica substrates were treated with 0.01% 3-aminopropyl triethoxysilane (Shin-Etsu Chemical Co., Ltd. Tokyo, Japan) to attach DNA-SWNTs to the frustule. The DNA-SWNTs in the frustule suspension were diluted 100 times with pure water. Then, 10 μL of the prepared sample was dropped onto the center of the mica substrate. After incubating for 20 min at room temperature, the sample was washed twice with 1 mL of pure water and dried at room temperature. Finally, structural analysis of the prepared samples was performed using AFM.

## Results and Discussion

**Figure 1:**
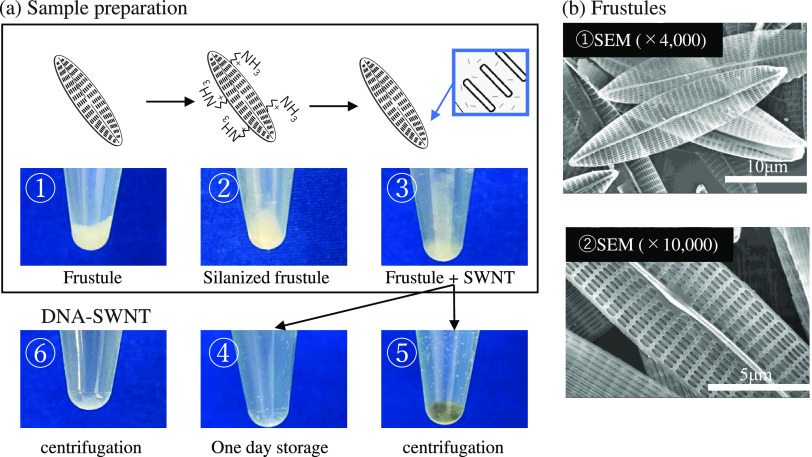
(a) Scheme of sample preparations. ① Purified bare frustules, ② frustules after salinization with APTES, ③ frustules attaching DNA-SWNT and frustules became gray color after removing DNA-SWNTs, ④ frustule suspensions after 1 day, ⑤ centrifugation of frustule suspensions after 1 day, and ⑥ DNA-SWNT suspensions. (b) Frustules observed by SEM. The length and width of frustules were almost 33.17 ± 0.97 and 7.35 ± 0.74 μm, respectively.

Figure 1a shows the schemes of our sample preparation with macroscopic images of fabricated micron-sized devices. DNA-SWNT suspension was prepared by a previously established method.^38^ SWNT powder was put into a DNA aqueous solution, and then, the mixture was sonicated to de-bundle SWNTs and attach DNA molecules on SWNT surfaces. The sample was centrifuged to remove aggregates, and supernatant was stored as DNA-SWNT hybrids. DNA-wrapped SWNTs do not precipitate to know it from this procedure.

Frustules, micron-sized nanoporous silica, were purified from cultivated living diatom cells. The size and shape were confirmed by SEM (Figure 1b). The length and width of diatom cells were almost 33.17 ± 0.97 and 7.35 ± 0.74 μm, respectively. The major pores were approximately 480 ± 30 and 140 ± 30 nm in length and width, respectively. In general, the specific gravity of frustules is around 1.3^36,37^ and that of silica beads is approximately 2.2–4.3.^39^ Because specific gravity of frustules is smaller than that of other typical micron-sized objects, they are widely used to make micron-sized biodevices. Frustules remain well suspended in solutions, yet they can easily be collected by gentle centrifugation. Their large surface area is also beneficial since it accelerates biological reactions on frustule surfaces.

To attach DNA-SWNT hybrids to frustules, frustules were pretreated with 1% APTES. Through silanization, the frustule surface can be functionalized with amino groups. When DNA-SWNT suspensions are injected into aminated frustule suspensions, DNA-SWNT hybrids bind to frustule surfaces via electrostatic interactions. DNA and amino groups have negative and positive charges, respectively, in neutral solutions. Because frustules can be collected by gentle centrifugation, excess APTES and DNA-SWNT hybrids can be removed by repeated centrifugation and removal of the supernatant.

The photographs in Figure 1a show the features of the frustules at each step. Purified bare frustules exhibited white soft precipitates when the suspension was stored for a long time (Photo ①). Even after silanization with APTES, there was no change in the frustules (Photo ②). When DNA-SWNT hybrids were mixed with the functionalized frustule suspension, the frustules became gray, even after the removal of excess DNA-SWNT hybrids (Photo ③). The obtained DNA-SWNT-attached frustules were used for subsequent evaluation.

Photographs ④ and ⑤ in Figure 1 show the easy collection of functionalized frustules by 1 day of storage or by centrifugation. Gray pellets were obtained in both cases. As a reference, the DNA-SWNT suspension without frustules was centrifuged under the same conditions (Photo ⑥). No aggregates or pellets were observed. DNA-SWNT hybrids could not be collected by gentle centrifugation.

Figure S1a shows a photograph of the DNA-SWNT frustule suspension without silanization. Without functionalization, the frustule surfaces were white, even after mixing with the DNA-SWNT suspension. This suggests that AP treatment was necessary to attach the DNA-SWNT hybrids to the frustule surfaces. Figure S1b,c shows the PL measurements without silanization. Without silanization, the DNA-SWNT frustule suspension showed no detectable PL. Therefore, silanization is an effective method for bonding DNA-SWNTs to frustule surfaces.

Figure S2 shows AFM images of frustules functionalized with DNA-SWNT hybrids. The frustule suspension was deposited on the AP–mica surface and observed using AFM in AC mode and phase mode in air. We confirmed that DNA-SWNTs adhered to the surface of the frustule.

Attachment of DNA-SWNT hybrids to aminated frustules was confirmed by absorbance measurement of the supernatants after centrifugation (Figure S3). After incubating the mixture of DNA-SWNTs and aminated frustules, the samples were centrifuged to remove unattached DNA-SWNTs and DNA molecules. DNA has an absorbance of only approximately 260 nm. Thus, the absorbance of the supernatants at 808 nm originated from SWNTs. The amount of SWNTs was very small (17% of the mixed amount), even in the supernatant of the first centrifugation. This suggests that many of the DNA-SWNTs were well attached to the functionalized frustule surfaces. The attachment of the SWNTs was confirmed by Raman spectroscopy (Figure S4). The presence of SWNTs on the frustules was indicated by Raman peaks. No change in the G and D bands of SWNTs was observed before and after attaching DNA-SWNTs to frustules.

**Figure 2:**
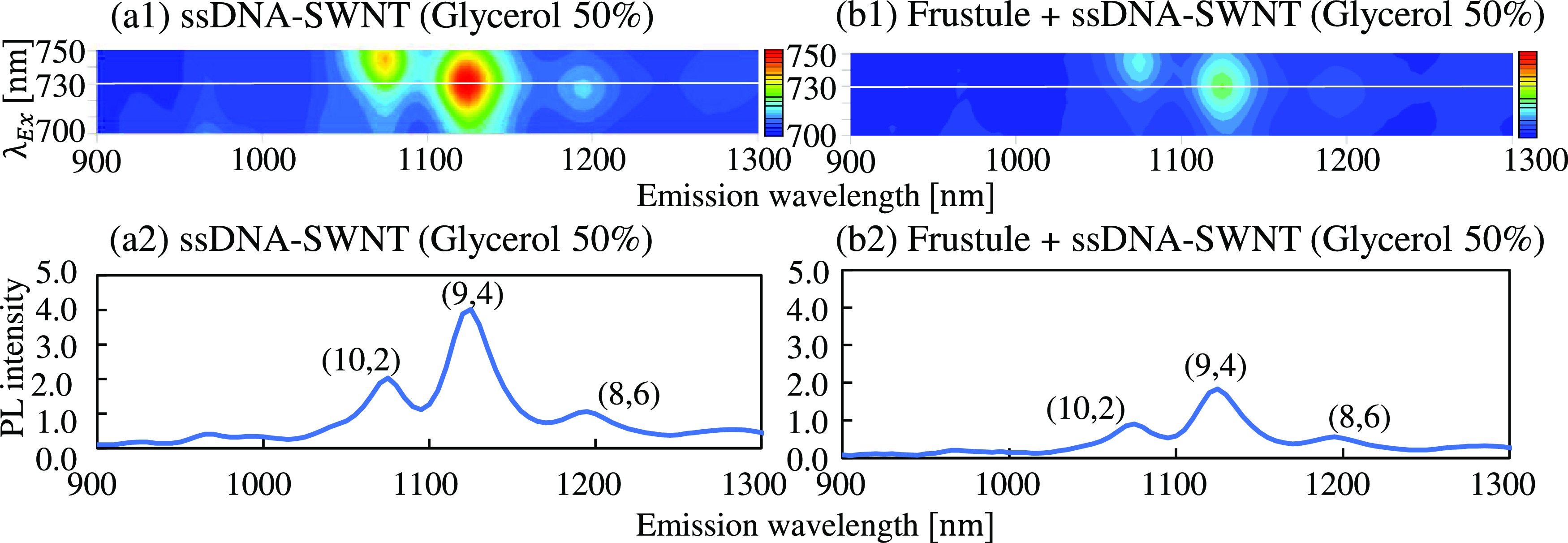
PL maps show (a1) ssDNA-SWNTs and (b1) ssDNA-SWNTs on frustules. The excitation and emission wavelength ranges were 700–750 and 900–1300 nm, respectively. Graphs are drawn for the cross-sections of the PL map at an excitation wavelength of 730 nm of the PL maps. These spectra show (a2) ssDNA-SWNTs and (b2) ssDNA-SWNT on frustules. Each suspension was 10 mM PBS buffer, including 50% glycerol. The absorbance of DNA-SWNT at 808 nm was adjusted to 0.2, and the turbidity of frustules at 750 nm was 0.5.

Figure 2 shows the PL maps and spectra of free DNA-SWNT hybrids and those attached to the aminated frustule surfaces. Because DNA and frustules do not have NIR PL, the observed PL spectra originated from the SWNTs. As shown in the PL maps, PL spots originating from three different SWNT chiralities were observed in both samples. For comparative purposes, the absorbance of the samples at 808 nm was adjusted to 0.2; thus, the same number of SWNTs was included in each sample. To avoid the settlement of frustules, 50% glycerol was added to the samples. When comparing the PL spectra with an excitation wavelength of 730 nm, the free DNA-SWNT hybrids revealed higher intensities. Table 1 lists the numerical values of the PL intensities of the measurements as an average of three independent samples. Interestingly, the ratios of PL intensity of free DNA-SWNTs to DNA-SWNT hybrids on frustules were 2.26, 2.18, and 1.88 for (10, 2), (9, 4), and (8, 6) chiralities, respectively. The effects of attachment on the PL intensity varied due to the SWNT chirality. In addition, the fifth supernatant obtained during the removal of excess DNA-SWNT hybrids by centrifugation was examined. No significant PL was observed (Figure S5). This suggests that the DNA-SWNTs that did not attach to frustule surfaces were negligible during the PL measurements, as shown in Figure 2a.

**Table 1:** PL Intensities of Three Chiralities in DNA-SWNTs and DNA-SWNT Frustules

	(10, 2)	(9, 4)	(8, 6)
	PL of peak	PL of peak	PL of peak
DNA-SWNTs	2.03 ± 0.13 (1075 nm)	4.01 ± 0.17 (1125 nm)	1.05 ± 0.06 (1195 nm)
DNA-SWNT frustules	0.90 ± 0.07 (1075 nm)	1.84 ± 0.09 (1125 nm)	0.56 ± 0.04 (1195 nm)

The absorbance of the DNA-SWNT frustules at 808 nm was adjusted to 0.2, and the turbidity of the DNA-SWNT frustules at 750 nm was 0.5.

**Figure 3:**
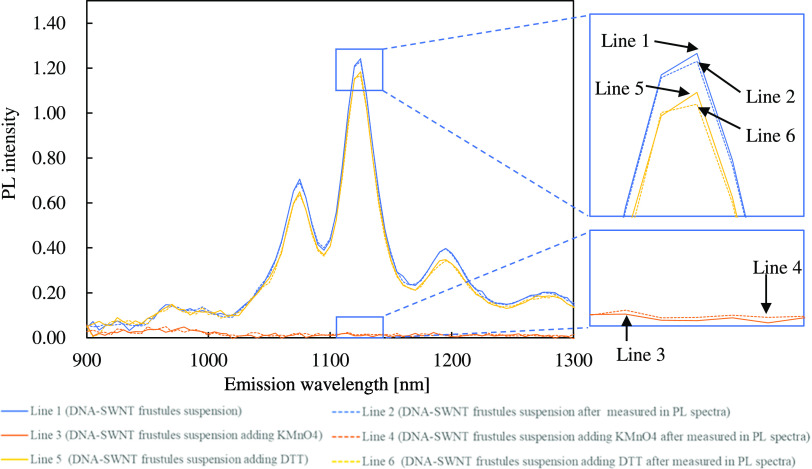
PL spectra at an excitation wavelength of 730 nm. Responses of SWNTs on frustules induced by addition of an oxidant and a reductant. Line 1 shows SWNTs on frustules. Line 2 is measured after Line 1. Line 3 shows SWNTs on frustules after adding an oxidant. Line 4 is measured after Line 3. Line 5 shows SWNTs on frustules after adding the oxidant and reductant. Line 6 is measured after Line 5. The absorbance of DNA-SWNT frustules at 808 nm was adjusted to 0.2, and the turbidity of DNA-SWNT frustules at 750 nm was 0.5. Oxidant is KMnO_4_ solution (final concentration: 5 μM), and reductant is DTT solution (final concentration: 50 μM).

To assess the potential of the fabricated DNA-SWNT frustules for use as biosensors, the PL responses of SWNTs on frustules induced by the addition of an oxidant and a reductant were examined. Previous reports have revealed that reducing and oxidizing agents induce drastic change of PL spectra when injected to a DNA-SWNT suspension.^4^ To achieve rapid diffusion, glycerol was not added to the samples at this instance (Figure 3). For this reason, we measured the PL spectra only by excitation at 730 nm to minimize the measurement period. To confirm the decrease in PL intensity due to the settlement of frustules, measurements were repeated twice for the same sample under the same conditions. Under these conditions, it took 45 s for each measurement; thus, the first and second measurements were carried out from 0 to 45 and 55 to 100 s, respectively. This would likely be accelerated if stirring of the suspension during measurements was possible.

Lines 1 and 2 in Figure 3 show the PL spectra of the first and second measurements of the same sample before the addition of the chemicals. The peak PL intensity of (9, 4) SWNTs was 1.24 and 1.23 for the first and the second measurements, respectively. The numerical values are summarized in Table 2. The difference between the first and second measurement indicates the effects of frustule settlement during the measurements. The frustules remained well suspended during the measurements without stirring. Then, 5 μL of KMnO_4_ (final concentration: 5 μM) was added and mixed into the sample. After incubating for 10 min at room temperature, the sample was shaken before the PL spectra were obtained again. Lines 3 and 4 (Figure 3) show the PL spectra in the presence of KMnO_4_ for the first and second measurements, respectively. It is clearly visible that the PL was completely quenched by KMnO_4_. Finally, 5 μL of DTT (final concentration: 50 μM) was added as a reductant and mixed into the sample. After incubating for 10 min at room temperature, the sample was shaken before the PL spectra were obtained again. Lines 5 and 6 show the PL spectra of KMnO_4_ and DTT, respectively. PL intensities of the first and second measurements were 1.18 and 1.17 for (9, 4) SWNTs, respectively. Recovery of PL intensity induced by the addition of DTT was 93% compared to the initial PL values without the chemicals. In the above numerical values, the effects of increasing sample volumes owing to the injection of the chemicals were appropriately revised. No PL peak shifts were observed. These experiments clearly indicate that the DNA-SWNT hybrids can be applied to biosensing as well as the usual DNA-SWNT suspension.

**Table 2:** PL Intensities of Three Chiralities in DNA-SWNT Frustules after Adding the Oxidant and Reductant^a^

			(10, 2)	(9, 4)	(8, 6)
		oxidant reductant	PL intensity	PL intensity	PL intensity
DNA-SWNT frustules			0.71 ± 0.03 (1075 nm)	1.24 ± 0.06 (1125 nm)	0.40 ± 0.02 (1195 nm)
DNA-SWNT frustules	KMnO_4_		0.02 ± 0.01 (1075 nm)	0.01 ± 0.01 (1125 nm)	0.01 ± 0.01 (1195 nm)
DNA-SWNT frustules	KMnO_4_	DTT	0.65 ± 0.02 (1075 nm)	1.18 ± 0.07 (1125 nm)	0.35 ± 0.02 (1195 nm)

a Absorbance of DNA-SWNT frustules at 808 nm was adjusted to 0.2, and turbidity of DNA-SWNT frustules at 750 nm was 0.5. Oxidant is KMnO_4_ solution (final concentration: 5 μM) and reductant is DTT solution (final concentration: 50 μM).

Catechin (final concentration: 50 μM) was used as an example of a biomolecule with antioxidant properties (Figure S6). Ishibashi et al. succeeded in detecting the antioxidant activity of catechins using the PL spectra of DNA-SWNT suspensions.^4^

Figure S6 shows the PL spectra at an excitation wavelength of 730 nm. The antioxidant ability of catechin molecules was successfully detected even with DNA-SWNT hybrids attached to frustule surfaces. This suggests that DNA-SWNT frustule devices have the potential to be applied to micron-sized biosensors.

**Figure 4:**
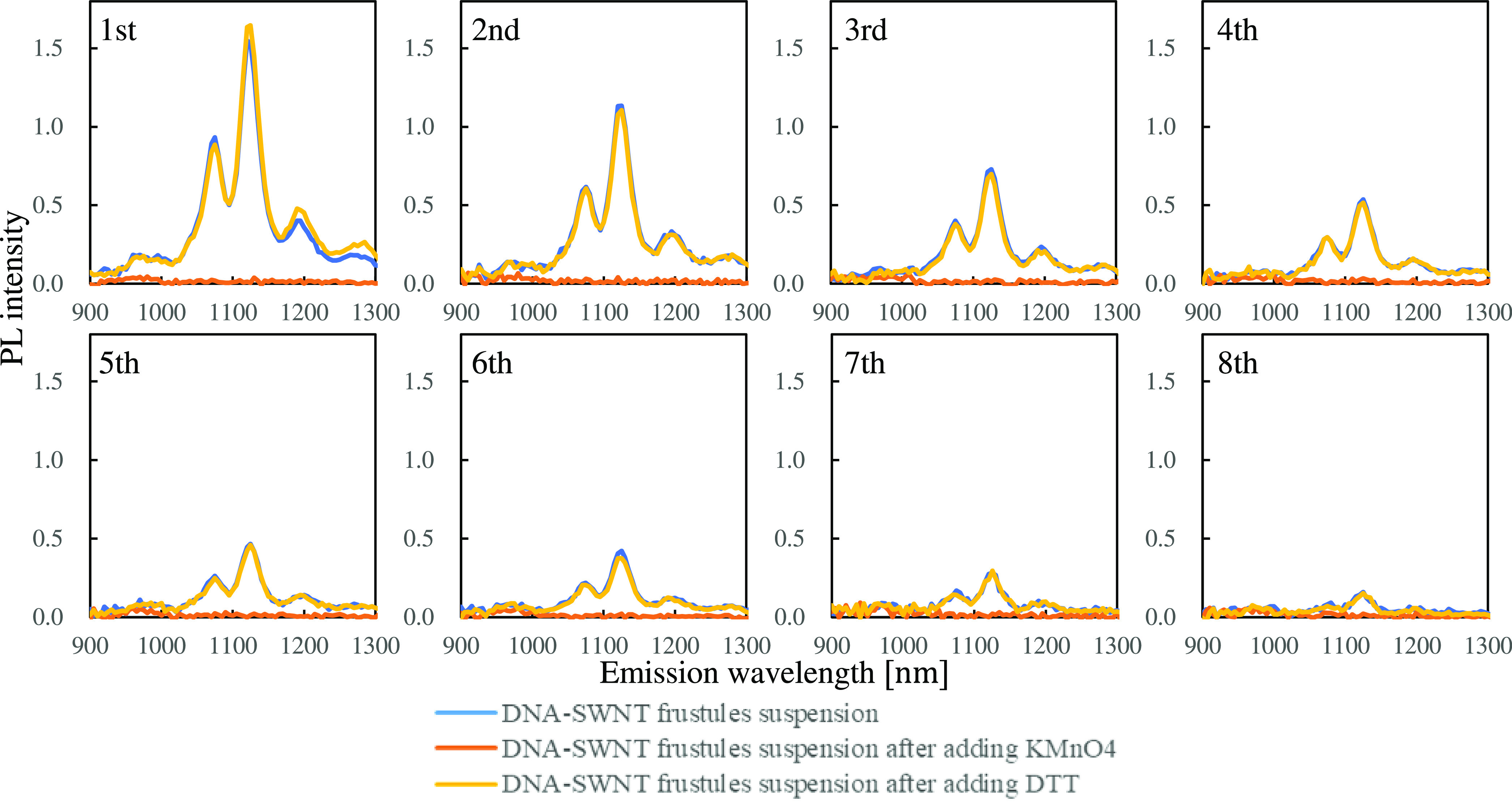
PL spectra at excitation wavelengths of 730 nm responses of SWNTs on frustules induced by addition of an oxidant and a reductant. Blue line shows initial PL spectra of DNA-SWNT frustules. The same suspension was then measured after injecting KMnO_4_ (final concentration: 5 μM) (orange line) and DTT (final concentration: 50 μM) (yellow line). The suspension was centrifuged at 5000*g* for 5 min, and then 80% of supernatant was replaced with a 5 mM PBS buffer. The centrifugation was repeated five times. The recovered DNA-SWNT frustule suspension was reused for the redox measurements. We repeated this cycle eight times. Measurements were performed thrice independently.

We evaluated the reusability of DNA-SWNT frustules. Figure 4 shows the PL spectrum of DNA-SWNT frustules at an excitation wavelength of 730 nm. The blue line shows the initial PL spectra of the DNA-SWNT frustules. The same suspension was then measured after injecting KMnO_4_ (orange line) as the oxidant. The same suspension was measured again after injecting DTT (yellow line) as the reductant. After the measurements, the suspension was recycled, and the oxidant and reductant were removed via centrifugation. This cycle was repeated eight times. Up to the seventh cycle, the (10, 2), (9, 4), and (8, 6) peaks showed redox reactions. In the eighth cycle, however, only the (9, 4) peak showed redox reactions.

## Conclusions

We proposed a micron-sized biodevice based on the PL of SWNTs attached to nanoporous biosilica. The micron-sized SWNT device exhibited sufficient PL response to detect redox reactions. In contrast to colloidal suspensions, micron-sized devices can be handled easily using gentle centrifugation. Recovery of the DNA-SWNT frustules was achieved by simple centrifugation, and the device could be reused.

## Supporting Information Available

Results of DNA-SWNT frustules without silanization; DNA-SWNTs on the frustule measured by AFM; absorbance of SWNTs in the supernatant after attaching SWNTs on the frustule surface; Raman spectra of DNA-SWNT frustules; PL of SWNTs in the supernatant from attaching SWNTs on the frustule surface; PL spectra of SWNTs on frustules induced by the addition of KMnO_4_ and a catechin (PDF)

## Abbreviations

PL: photoluminescence
SWNTs: single-walled carbon nanotubes
CNTs: carbon nanotubes
APTES: 3-(2-aminoethylaminopropyl) trimethoxysilane
DTT: dithiothreitol
PBS: phosphate-buffered saline
