# Supplementary material for: Attachment of DNA-Wrapped Single-Walled Carbon Nanotubes (SWNTs) for a Micron-Sized Biosensor
Source: ACS Omega. 2022 Dec 9;7(50):47148–55. doi: 10.1021/acsomega.2c06278 (PMC9774338; doi:10.1021/acsomega.2c06278)
Supplement: Supplementary file 1 — ao2c06278_si_001.pdf [file ao2c06278_si_001.pdf]

Supporting Information

Attachment of DNA wrapped single-walled carbon nanotubes (SWNTs) for a micron size biosensor

Kota Hirayama[1], Masaki Kitamura[1], Nay San Lin[1], Minh Hieu Nguyen[2], Binh Duong Le[3], Anh Tuan Mai[4], Shigeki Mayama[5], Kazuo Umemura[1*]

[1]Biophysics Section, Department of Physics, Faculty of Science Division II, Tokyo University of Science, 1-3 Kagurazaka, Shinjuku, Tokyo 162-8601, Japan
[2]VNU University of Science, 334 Nguyen Trai, Thanh Xuan, Hanoi, Vietnam
[3]National center for Technological progress, 25 Le Thanh Tong, Hoan Kiem, Hanoi, Vietnam
[4]VNU University of Engineering and Technology, 144 Xuan Thuy, Cau Giay, Hanoi, Vietnam
[5]Tokyo Diatomology Lab, 2–3-2 Nukuikitamachi, Koganei, Tokyo 184-00 15, Japan

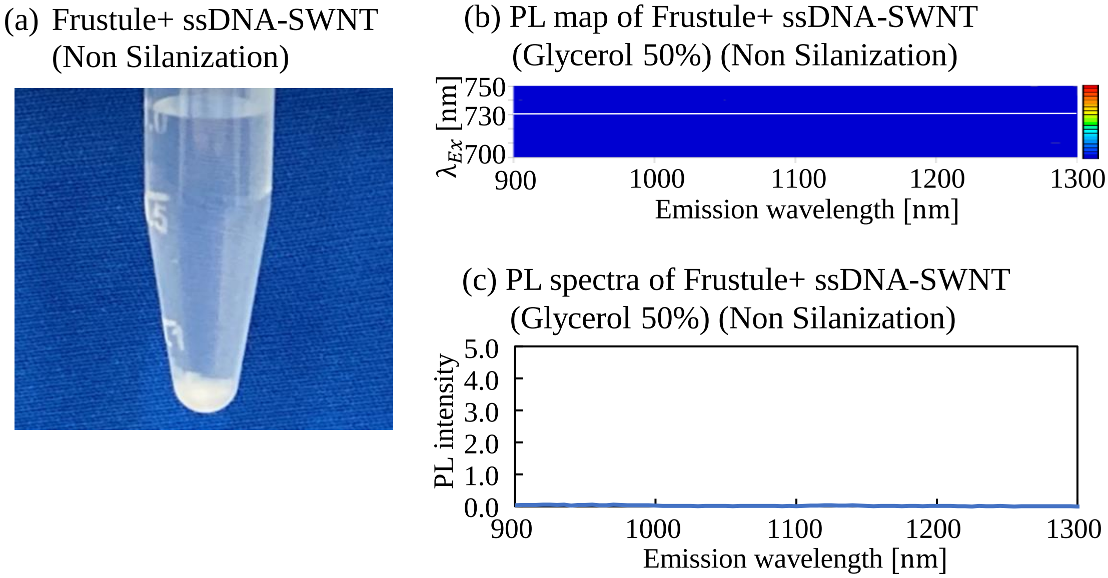

Figure S1 Frustules and DNA-SWNTs are mixed without silanization. (a) Photo of frustule ＋ DNA-SWNT (b) PL map. The excitation and emission wavelength ranges were 700–750 nm and 900–1300 nm, respectively. (c) PL spectra at an excitation wavelength of 730 nm.

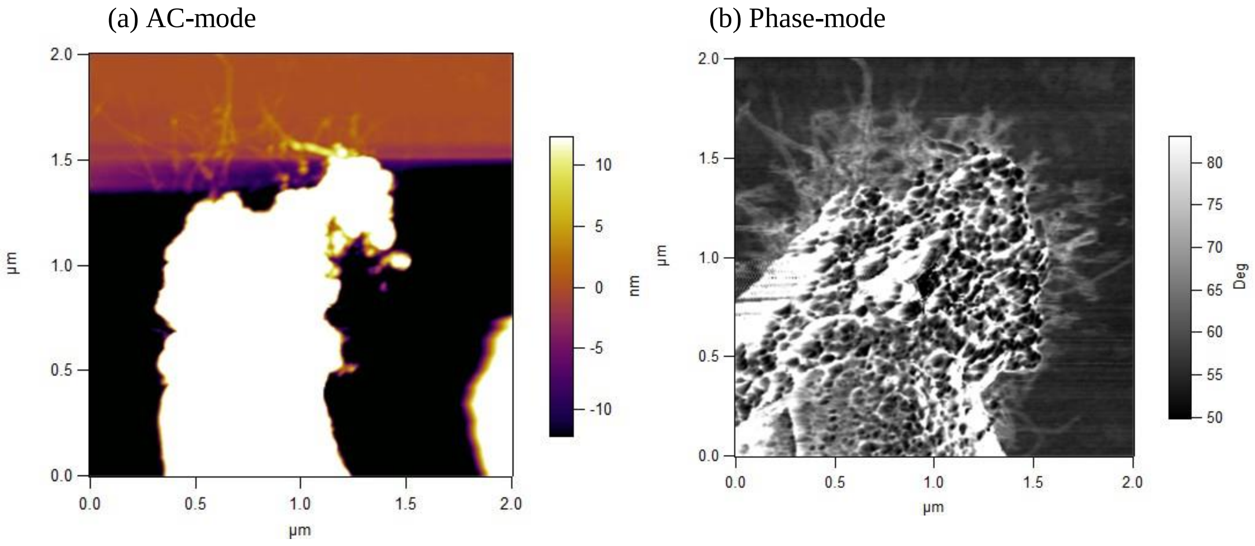

Figure S2 DNA-SWNTs on the frustule measured by AFM. Both (a) and (b) were measured at the same point on the frustule surface (a) Height adjusted to SWCNT in AC-mode (b) Phase-mode.

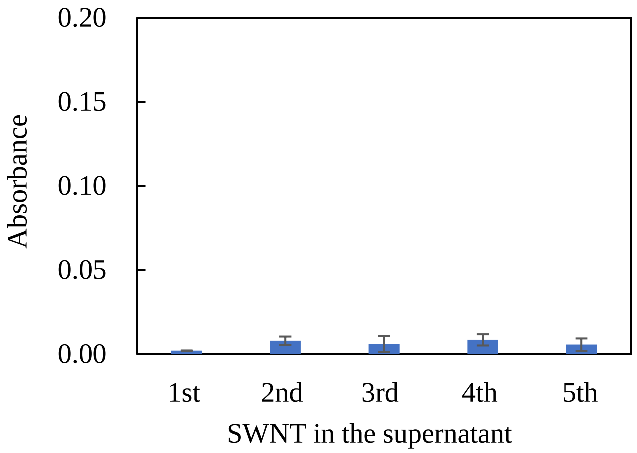

Figure S3 Absorbance of SWNTs in the supernatant from five centrifugations after attaching SWNTs on frustule surface. We measured five times.

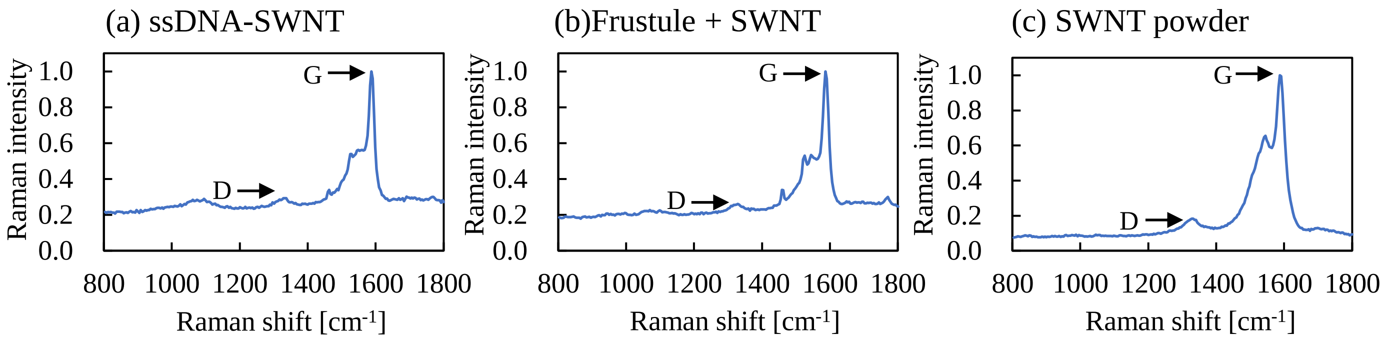

Figure S4 Raman spectra in the G band and D band (a) ssDNA wrapped SWNT (b) Frustule attaching SWNT (c) SWNT powder.

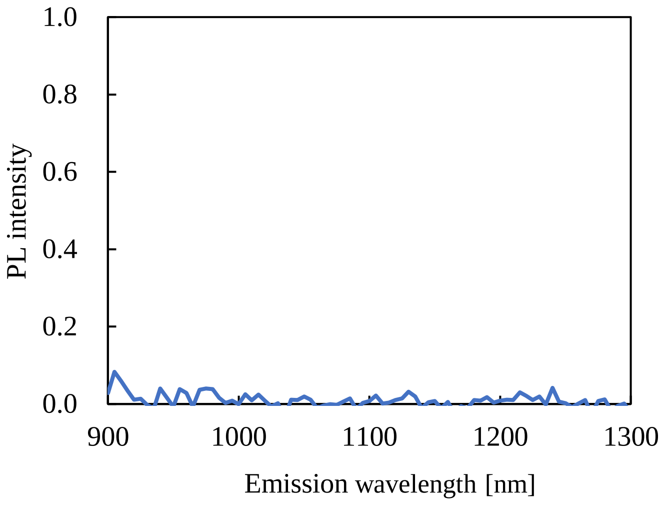

Figure S5 PL of SWNTs in the supernatant from first centrifugation after attaching frustule surface and SWNTs. Centrifugation of the samples were performed five times independently.

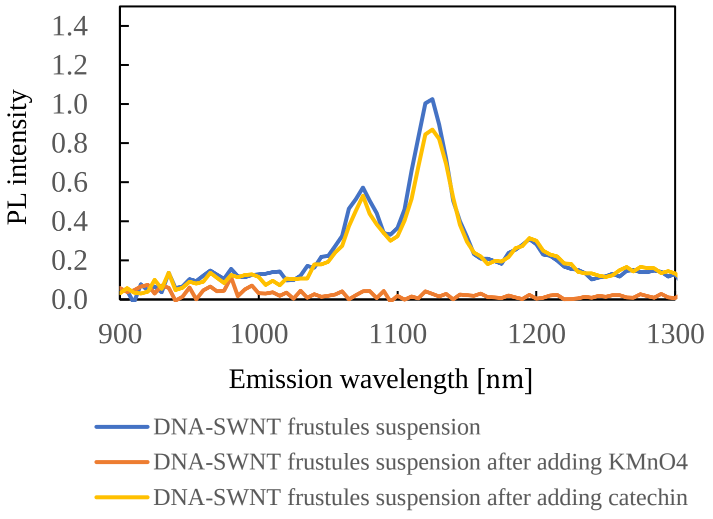

Figure S6 PL spectra at an excitation wavelength of 730 nm. Responses of SWNTs on frustules induced by the addition of an oxidant and a reductant. The blue line shows initial PL spectra of DNA-SWNT frustules. The same suspension was then measured after injecting $KMnO_4$ (final concentration: 5 μM) (Orange line) and catechin (final concentration: 50 μM) (Yellow line). Absorbance of DNA-SWNT frustules at 808 nm was adjusted to 0.2, and the turbidity of DNA-SWNT frustules at 750 nm was 0.5. Oxidant is $KMnO_4$ solution (final concentration: 5 μM), and reductant is catechin solution (final concentration: 50 μM). Measurements were performed independently thrice.
